# Dynamic Metabolic Disruption in Rats Perinatally Exposed to Low Doses of Bisphenol-A

**DOI:** 10.1371/journal.pone.0141698

**Published:** 2015-10-30

**Authors:** Marie Tremblay-Franco, Nicolas J. Cabaton, Cécile Canlet, Roselyne Gautier, Cheryl M. Schaeberle, Fabien Jourdan, Carlos Sonnenschein, Florence Vinson, Ana M. Soto, Daniel Zalko

**Affiliations:** 1 UMR1331, TOXALIM, Research Centre in Food Toxicology, Institut National de la Recherche Agronomique, INRA, Université de Toulouse, Toulouse, France; 2 Department of Integrative Physiology & Pathobiology, Tufts University School of Medicine, Boston, Massachusetts, United States of America; Deakin School of Medicine, AUSTRALIA

## Abstract

Along with the well-established effects on fertility and fecundity, perinatal exposure to endocrine disrupting chemicals, and notably to xeno-estrogens, is strongly suspected of modulating general metabolism. The metabolism of a perinatally exposed individual may be durably altered leading to a higher susceptibility of developing metabolic disorders such as obesity and diabetes; however, experimental designs involving the long term study of these dynamic changes in the metabolome raise novel challenges. ^1^H-NMR-based metabolomics was applied to study the effects of bisphenol-A (BPA, 0; 0.25; 2.5, 25 and 250 μg/kg BW/day) in rats exposed perinatally. Serum and liver samples of exposed animals were analyzed on days 21, 50, 90, 140 and 200 in order to explore whether maternal exposure to BPA alters metabolism. Partial Least Squares-Discriminant Analysis (PLS-DA) was independently applied to each time point, demonstrating a significant pair-wise discrimination for liver as well as serum samples at all time-points, and highlighting unequivocal metabolic shifts in rats perinatally exposed to BPA, including those exposed to lower doses. In BPA exposed animals, metabolism of glucose, lactate and fatty acids was modified over time. To further explore dynamic variation, ANOVA-Simultaneous Component Analysis (A-SCA) was used to separate data into blocks corresponding to the different sources of variation (Time, Dose and Time*Dose interaction). A-SCA enabled the demonstration of a dynamic, time/age dependent shift of serum metabolome throughout the rats’ lifetimes. Variables responsible for the discrimination between groups clearly indicate that BPA modulates energy metabolism, and suggest alterations of neurotransmitter signaling, the latter finding being compatible with the neurodevelopmental effect of this xenoestrogen. In conclusion, long lasting metabolic effects of BPA could be characterized over 200 days, despite physiological (and thus metabolic) changes connected with sexual maturation and aging.

## Introduction

A variety of anthropogenic compounds act as endocrine disrupting chemicals (EDCs). Adverse effects of EDCs have been documented in humans, in wildlife and in laboratory experiments [1]. The effects observed in animal models parallel the increase of similar pathologies such as obesity, neurobehavioral deficits and breast cancer observed in humans. This is particularly true for xeno-estrogens (and anti-androgens), when exposure occurs during development. Indeed, low doses of EDCs produced adverse effects when exposure occurred during specific windows of sensitivity, particularly during early fetal development [2, 3]. In addition to reproductive alterations in rodents of both genders, perinatal exposure studies using DES and bisphenol-A (BPA) as model xeno-estrogens, have unveiled a broader range of deleterious effects on behavior, thyroid and cardiovascular endocrinology [1, 4], even at low doses of exposure [5]. Of particular note, xeno-estrogens modulate the metabolism, and could contribute to the onset of obesity [6, 7].

BPA is a major endocrine disruptor which is used in the production of polycarbonate plastics and epoxy resins. BPA-based polymers can release free BPA under extreme conditions of heat and/or pH, and human exposure can occur through different routes [8]. Although BPA does not bioaccumulate, pregnant women and newborns are almost continuously exposed to low doses of this EDC [9–11]. It has been reported that BPA induces metabolic changes in animals exposed during the perinatal period [3, 12]. However, unveiling the metabolic effects of low-dose exposures requires the implementation of methods allowing for the simultaneous analysis of multiple metabolic targets. The use of non-targeted methodologies, and more specifically of metabolomics, is a very promising approach in this regard. Moreover, combining metabolomics with the bioinformatic modeling of the functional metabolic network is expected to pave the way for a more precise understanding of the altered metabolic modulation triggered by EDC, which needs to be thoroughly characterized for model compounds.

Recently, we showed that subtle changes in the global metabolism of mice resulting from perinatal exposure to BPA could be characterized using ^1^H-NMR metabolomics [13]. More specifically, we explored BPA effects at postnatal days (PND) 2 and 21. Using NMR-based metabolomics, based on the spectrometric analysis of serum, liver or brain, we were able to discriminate all treated groups (low-dose as well as very-low-dose exposed animals) from controls. However, these studies were not designed to examine the occurrence of long-lasting effects, nor did they address the possibility of exploring time/dose interactions for BPA. Although longitudinal metabolomic studies could theoretically be carried out in rodents over several months using serum or urine samples, such studies become unfeasible and potentially inhumane when aimed at including direct characterization of metabolic modulations at the hepatic level, based on repeated liver sampling. For this reason, multiple time-point studies that rely on multiple groups of animal must be used, which though common in toxicological studies, requires the development of specific statistical approaches in the field of metabolomics and systems biology. To do this, we extended our previous study in mice [13] to a study using multiple doses (5) and time points (5) in rats up to PND200, to address the lasting metabolic consequences of perinatal exposure to low doses of BPA, and examined those effects despite physiological changes related to puberty and aging.

Metabolomic studies rely on spectral analyses of biofluids or tissue extracts, followed by multivariate statistics seeking to discriminate between the different experimental groups. PCA or PLS-DA are routinely used to analyze metabolomic data when examining multiple groups of animals and time points, and provide a powerful option for a given time point or dose. Unfortunately, these methods take into account neither the dependence nor the temporal structure of data. Multivariate ANOVA, the most frequently used method to analyze experimental data, is unsuitable to evaluate data obtained from longitudinal metabolomic studies, especially when the sample size is much smaller than the total number of variables. Therefore, a major part of the information is lost when either ANOVA or PCA are used alone to analyze temporal changes in the metabolome. The ANOVA-Simultaneous Component Analysis method (A-SCA, [14–16]), which combines an ANOVA and a PCA step, provides an alternative yet complementary approach which enables the independent analysis of the effect of each experimental factor (time, dose,…).

In the current study, we evaluated the dynamic effects of low doses of BPA on Sprague-Dawley (SD) rat offspring that were perinatally exposed to BPA. Namely, fetuses and neonates were exposed to low doses of BPA (0.25μg, 2.5μg, 25μg or 250μg BPA/kg BW/day) administered to their mothers from gestational day (GD) 9 through day 16 of lactation. Samples of liver (the main metabolizing organ) and serum (circulating metabolites) from female offspring were collected at postnatal day (PND) 21 (weaning), 50 (mature adults), 90, 140 and 200 (older adults). Based on NMR data, we first applied classical PLS-DA analyses to confirm previous findings in mice and to explore lasting long term effects. In a second step, we applied A-SCA to further explore the respective contribution of the Dose, Time, and Dose*Time interactions. Finally, we analyzed the data in the context of the functional metabolic network to gain a better understanding of the metabolic pathways modulated by low doses of BPA exposure.

## Materials and Methods

### Chemicals

Dimethyl-sulfoxide (DMSO, CAS #67-68-5), bisphenol-A (4,4'-dihydroxydiphenyl-dimethylmethane, CAS #80-05-7, product #239658, lot #03105ES; purity ~99%) and butylated-hydroxytoluene (BHT) were purchased from Sigma Chemical Company (Saint Louis, MO). BPA stock purity was confirmed as described previously [4]. Acetonitrile was purchased from Scharlab SL (Sentmenat, Spain), deuterium oxide (D_2_O) and sodium 3-trimethylsilyl-2,2,3,3-tetradeuteriopropionate (TMSP) from Euriso-top (Saint-Aubin, France).

### Animals

Sexually mature virgin female SD rats (8–10 weeks of age, Taconic, Germantown NY) were maintained in temperature and light controlled (14/10 hr light/dark cycles) conditions at the Tufts University School of Medicine Division of Laboratory Animal Medicine. Experimental procedures were approved by the Tufts University & Tufts Medical Center Institutional Animal Care and Use Committee (IACUC) and all animals were treated humanely in accordance with the Guide for Care and Use of Laboratory Animals. Cages, water bottles and bedding tested negligible for estrogenicity by the ESCREEN assay [17]. Food (Harlan Teklad 2018) was supplied *ad libitum*. Estrogenicity of the feed was measured at 8–15fmoles of estrogen equivalents per gram, a negligible amount. Female rats were mated with SD males. The morning on which sperm was observed in vaginal smears was designated gestational day (GD) 1. Dams (N = 9-12/dose/exposure period) were subcutaneously implanted on day 9 of pregnancy with Alzet osmotic pumps (cat#2004, Durect Corp., Cupertino, CA) designed to deliver continuously up to 28 days to administer vehicle (50% dimethyl sulfoxide) or 0.25, 2.5, 25, or 250 μg BPA/kg BW/d. The dose was calculated based on the weight of the dam at day 7 of pregnancy. For convenience, these doses are subsequently referred to as Control, BPA0.25, BPA2.5, BPA25, or BPA250. Animals delivered normally and litters were culled to 10 individuals on PND2. All female offspring (N = 9-12/dose/age at sacrifice/exposure period) were distributed so that each litter was represented only once. At each time point (e.g. PND 21, 50, 90, 140 and 200), one F1 female rat that had been exposed perinatally to either vehicle or BPA was randomly chosen from each litter and euthanized (CO_2_ euthanasia). Blood and liver were collected.

### Sample preparation and ^1^H-Nuclear Magnetic Resonance (NMR) spectroscopy

Serum samples were centrifuged for 5min at 10,000g and 20°C. Serum was collected in microtubes and stored at -20°C. Serum samples (100μL) were diluted with 600μL of D_2_O and centrifuged at 5,000g for 10min before they were placed in 5mm NMR tubes. Liver extractions were performed as described previously [13]. Briefly, samples of liver (100mg) were homogenized using a Polytron PT2100 in acetonitrile/H_2_O (50/50, v/v) containing 0.1% BHT in an ice-water bath. Homogenates were centrifuged at 5000g for 10min at 4°C, and the supernatants were removed and lyophilized. Lyophilisates were then reconstituted in 600μl of D_2_O containing 0.25 nM TMSP, as a chemical reference at 0 ppm. The reconstituted samples were transferred into NMR tubes.

All ^1^H-NMR spectra were obtained on a Bruker DRX-600-Avance NMR spectrometer operating at 600.13 MHz for ^1^H resonance frequency using an inverse detection 5mm ^1^H-^13^C-^15^N cryoprobe attached to a CryoPlatform (the preamplifier cooling unit). The ^1^H-NMR spectra were acquired at 300K using the Carr-Purcell-Meiboom-Gill (CPMG) spin-echo pulse sequence with pre-saturation, as described previously [13], with a total spin echo delay (2nτ) of 240ms to attenuate broad signals from proteins and lipoproteins. A total of 128 transients were collected in 32,000 data points using a spectral width of 12 ppm, a relaxation delay of 2.5 sec, and an acquisition time of 2.28 sec. The spectra were Fourier transformed by multiplication of the FIDs by an exponential weighting function corresponding to a line-broadening of 0.3Hz. All spectra were manually phased and baseline corrected and referenced to TMSP using Bruker TopSpin 2.1 software (Bruker, GMBH, Karlsruhe, Germany). To confirm the chemical structure of metabolites of interest, 2D ^1^H-^1^H COSY (Correlation Spectroscopy) and 2D ^1^H-^13^C-HSQC (Heteronuclear Single Quantum Coherence Spectroscopy) NMR experiments were performed on selected samples. Spectral assignment was based on matching 1D and 2D data to reference spectra in a home-made reference database, as well as with other databases (http://www.brmb.wisc.edu and http://www.hmdb.ca), and reports in the literature.

### Data reduction and multivariate statistical analyses

Data were reduced using the AMIX software (version 3.9, Bruker, Rheinstetten, Germany) to integrate 0.01 ppm wide regions corresponding to the δ 9.0–0.70 ppm region for serum samples and to the 10.0–0.80 ppm region for aqueous liver extracts, respectively. The 5.10–4.40 and 5.0–4.50 ppm regions, comprising the water resonances for serum samples and aqueous liver extracts, respectively, were excluded. The 3.7–3.6 ppm and 1.25–1.10 ppm regions, corresponding to ethanol resonances, were also excluded for serum samples. A total of 738 (serum) and 871 (liver) NMR buckets were included in the data matrices. To account for differences in sample amount, each integrated region was normalized to the total spectral area. Measurements were performed on 6 to 10 rats per treatment group and per measurement time-point. Rats were nested both within treatment group and within measurement time point. The full design of the experiment is provided in S1 Fig.

In a first step, PLS-DA was used to model the relationship between BPA doses and spectral data for each time point. Before analysis, Orthogonal Signal Correction (OSC) filtering [18] was used to remove variation not linked to the treatment (e.g. physiological, experimental or instrumental variation). Filtered data were mean-centered and scaled (Unit or Pareto). The R^2^ and Q^2^ criteria were used to assess models performance. R^2^ quantifies the percentage of explained variance and Q^2^ the predictive capacity of the model. A robust model should be characterized by a R^2^ > 50% and a Q^2^ > 40% [19]. In addition, the robustness of our models was post-validated using permutation tests involving 200 iterations.

In a second step, and in order to further take into account the variation over time and the relationship between variables (multivariate data), the A-SCA method was applied to study the effect of Time and Dose on the metabolome [14–16]. A-SCA was developed to analyze metabolomic datasets taking into consideration the underlying experimental design. A-SCA combines ANOVA and PCA and works in two steps. ANOVA was first applied to split data according to the experimental factors, namely Time, Dose, and the Time*Dose interaction, as follows (Eq 1):

$$X=X_{Time}+X_{Dose}+X_{Time*Dose}+X_{Residual}.$$ Eq 1

A permutation test [20] was used to check the significance of the experimental factors of Eq 1(10,000 iterations). Next, each data block (*i*.*e*. each sub-model) was individually analyzed using PCA.

Discriminant variables were determined using loadings or VIP (Variable Importance in the Projection), for A-SCA and PLS-DA, respectively. Then, the Kruskal-Wallis test (significance threshold = 0.05) was applied to assess the significance of the difference. The Kruskal–Wallis test is a non-parametric method for comparing two or more groups [21]. When rejecting the null hypothesis of the test, pairwise comparisons were performed to identify which pairs were significantly different. Multiple testing corrections were used to avoid false positives. SIMCA-P+ software (V13, Umetrics AB, Umea, Sweden) and MATLAB (Mathworks, Inc., Natick, MA) were used to perform the multivariate analyses. A-SCA and permutation tests were performed according to the MATLAB functions from Jansen et al. (2005) and Vis et al. (2007) [14, 20]. Using this software available at http://www.bdagroup.nl., the original function estimates were obtained for the Time, the Dose and the Time*Dose interaction, respectively, as detailed in Eq 1. Since the focus of the study was on BPA effects (e.g., to separate the Dose factor), we modified the original MATLAB function to enable a distinction between the Dose factor and the interaction effect. In addition, an in-house MATLAB function was written to search sets of time points and BPA doses for which the Time*Dose interaction was significant. At least 3 time points were included in this set. A-SCA and permutation tests were applied for all combinations of k time points (k = 3 to 5) and h doses (h = 2 to 5).

Metabolomics datasets generated from either biofluids or tissues include hundreds of endogenous metabolites. Through an integrative approach, metabolic profiling provides an instantaneous picture of dynamic integrative processes (functional biochemical pathways). In addition, specific bioinformatic tools and methods such as the web-server MetExplore (www.metexplore.fr) have made it possible to “plug” the metabolite shifts into a global metabolic network to produce a very informative picture of the status of the system while opening the way for the interpretation of the observed changes [22]. In the last stage of the study, we attempted to connect discriminant metabolites through relevant cascades of metabolic reactions. This computation was based on the *Rattus norvegicus* metabolic network [23] which currently comprises 890 reactions. In order to focus on a relevant subset of reactions we first computed a sequence of connecting reactions for each pair of biomarkers [24, 25]. All these sequences were gathered in a single network containing a few dozen reactions, allowing for the retrieval of biological information using visual mining. Graphical representation was obtained using the MetExplore web server, in combination with Cytoscape [22, 26].

## Results

**Fig 1:**
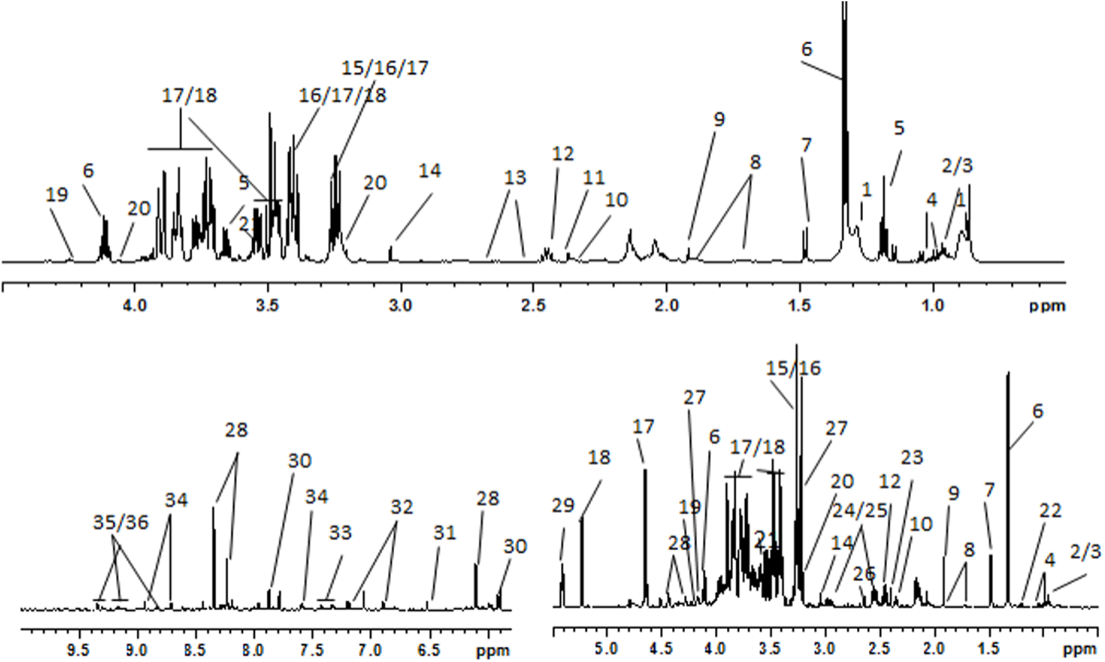
Typical 600 MHz ^1^H-NMR spectra from a control rat. A) Serum sample. B) Aqueous liver extract sample. *Peak 1*, low-density-lipoproteins/very-low-density-lipoproteins (LDL/VLDL); *peak 2*, leucine; *peak 3*, isoleucine; *peak 4*, valine; *peak 5*, ethanol; *peak 6*, lactate; *peak 7*, alanine; *peak 8*, lysine; *peak 9*, acetate; *peak 10*, glutamate; *peak 11*, pyruvate; *peak 12*, glutamine; *peak 13*, citrate; *peak 14*, creatine; *peak 15*, betaine; *peak 16*, taurine; *peak 17*, β-glucose; *peak 18*, α-glucose; *peak 19*, threonine; *peak 20*, choline; *peak 21*, glycine; *peak 22*, 3-hydroxybutyrate; *peak 23*, succinate; *peak 24*, reduced glutathione (GSH); *peak 25*, oxidized glutathione (GSSG); *peak 26*, hypotaurine; *peak 27*, phosphorylcholine; *peak 28*, inosine; *peak 29*, glycogen; *peak 30*, uridine; *peak 31*, fumarate; *peak 32*, tyrosine; *peak 33*, phenylalanine; *peak 34*, niaciamide; *peak 35*, NAD+; *peak 36*, NADP+.

With the aim of identifying the metabolic fingerprints characteristic of perinatal low dose BPA exposure, high-resolution ^1^H-NMR spectra were recorded from serum samples and aqueous liver extracts (Fig 1). Twenty-seven and thirty-two metabolites were identified in serum samples and aqueous liver extracts, respectively, based on 1 and 2 Dimensional NMR spectra (S1 Table).

### PLS-DA analysis, liver and serum samples

**Fig 2:**
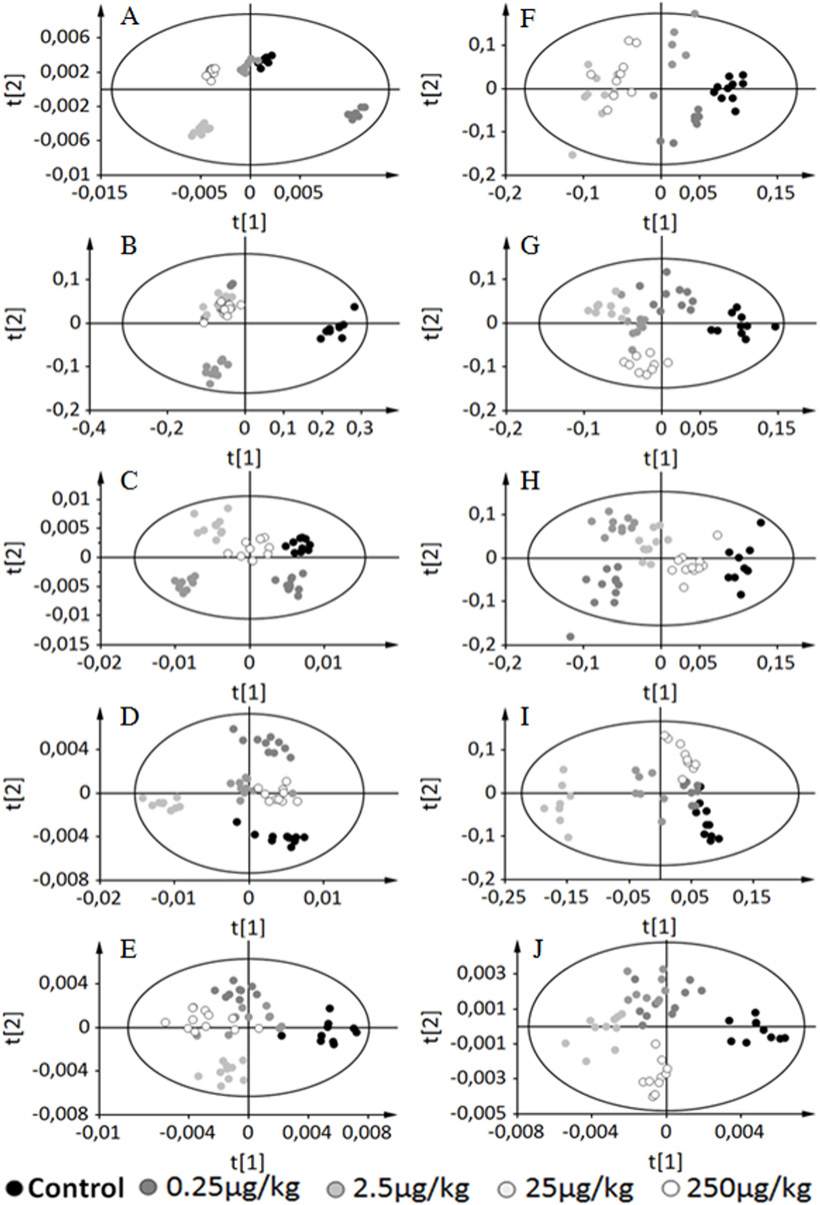
Two-dimensional PLS-DA scores plot of F1 samples integrated ^1^H-NMR spectra for each time point independently. Left column: Serum samples; Right column: Liver extracts. Each dot represents an observation (animal), projected onto first (horizontal axis) and second (vertical axis) PLS-DA variables. BPA doses are shown in different colors: Control in black, BPA0.25 colored dark grey, BPA2.5 in middle grey, BPA25 in light grey and BPA250 in white. The black ellipse determines the 95% confidence interval, which is drawn using Hotelling's T^2^ statistic. A. PND21 serum samples (A = 2, R^2^ = 49.4, Q^2^ = 0.484). B. PND50 serum samples (A = 3, R^2^ = 69.7, Q^2^ = 0.644). C. PND90 serum samples (A = 2, R^2^ = 47.0, Q^2^ = 0.449). D. PND140 serum samples (A = 2, R^2^ = 45.7, Q^2^ = 0.429). E. PND200 serum samples (A = 2, R^2^ = 42.5, Q^2^ = 0.378). F. PND21 aqueous liver extracts (A = 3, R^2^ = 49.5, Q^2^ = 0.277). G. PND50 aqueous liver extracts (A = 3, R^2^ = 80.0, Q^2^ = 0.504). H. PND90 aqueous liver extracts (A = 3, R^2^ = 63.0, Q^2^ = 0.517). I. PND140 aqueous liver extracts (A = 3, R^2^ = 58.2, Q^2^ = 0.357). J. PND200 aqueous liver extracts (A = 2, R^2^ = 43.4, Q^2^ = 0.396).

**Fig 3:**
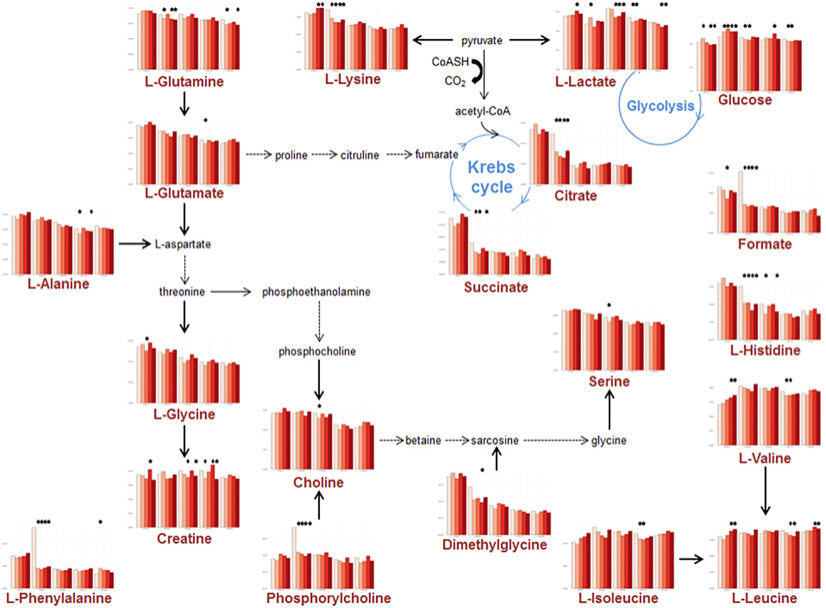
Schematic presentation of the discriminant metabolites in the serum samples. Bar graphs represent the average concentration of serum metabolites for all time-points and doses. For each metabolite 5 blocks are displayed corresponding (from left to right) to PND 21, 50, 90, 140 and 200, respectively. In each of these blocks, colors refer to the doses (white: control; pink: BPA0.25; light red: BPA2.5; red: BPA25; dark-red: BPA250). Stars indicate a significant difference (p-value < 0.05) between the Control group and the BPA group immediately underneath.

**Fig 4:**
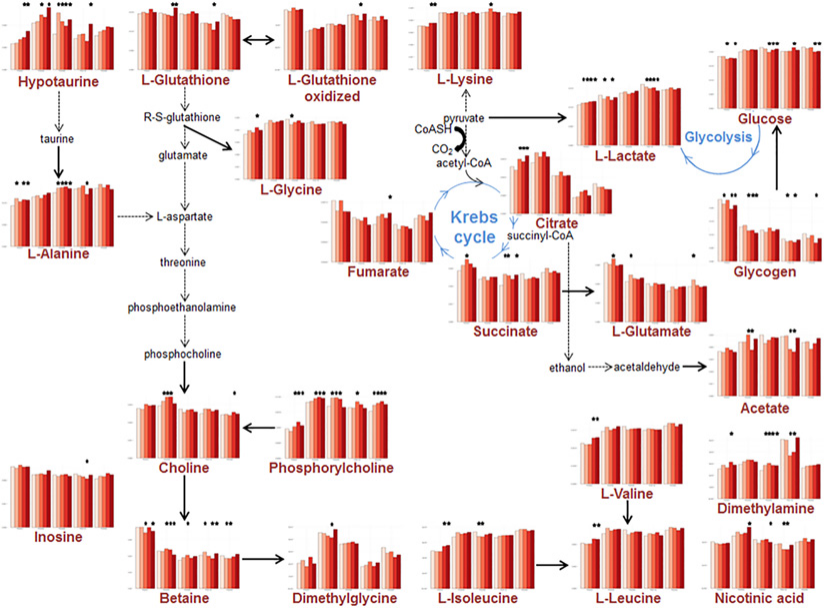
Schematic presentation of the discriminant metabolites in the Liver extract samples. Bar graphs represent the average concentration of liver metabolites for all time-points and doses. For each metabolite 5 blocks are displayed corresponding (from left to right) to PND 21, 50, 90, 140 and 200, respectively. In each of these blocks, colors refer to the doses (white: control; pink: BPA0.25; light red: BPA2.5; red: BPA25; dark-red: BPA250). Stars indicate a significant difference (p-value < 0.05) between the Control group and the BPA group immediately underneath.

We first applied PLS-DA to the full sets of data. As expected when combining 5 doses and 5 time points spread out over 200 days, the resulting models for serum and liver extracts were not valid, although all PND21 animals could tentatively be separated regardless of the dose (S2 Fig). Next, PLS-DA models were constructed for each time point separately based on the 5 doses taken together (controls + 4 BPA doses). Additionally, pairwise comparisons were carried out at each time-point. All 5-group PLS-DA models were valid and robust, as demonstrated by their respective R^2^ (percentage of explained variance) and Q^2^ (predictive capacity of the model) values (Table 1, a robust model should be characterized by a R^2^ > 50% and a Q^2^ > 40%). All PLS-DA models for pairwise comparisons were valid as well, with the exception of Controls (DMSO) *vs*. BPA 2.5 μg/kg exposed animals, at PND21 for serum samples. The corresponding two-dimensional score plots are displayed in Fig 2, and the discriminating variables (Figs 3 and 4, for serum and liver extracts respectively) are metabolites comprised of amino-acids, glucose, lactate and lipids. The relative concentration of these variables was significantly modified by perinatal exposure to low as well as very low doses of BPA.

**Table 1:** Pairwise PLS-DA model parameter comparisons.

		SERUM SAMPLES						LIVER EXTRACTS					
						Permutation test^*e*^						Permutation test^*e*^	
Time point	Dataset	N^*a*^	PLS components^*b*^	R2 (%)^*c*^	Q2^*d*^	R2 intercept	Q2 intercept	N^*a*^	PLS components^*b*^	R2 (%)^*c*^	Q2^*d*^	R2 intercept	Q2 intercept
PND 21	5 treatment groups	42	2	49.4	0.484	0.086	-0.156	43	3	49.5	0.277	0.267	-0.316
	Control / 0.25	16	1	99.2	0.771	0.249	-0.168	17	2	97.2	0.626	0.720	-0.217
	Control / 2.5	16	1	49.4	-0.10	0.515	0.006	16	1	97.7	0.530	0.100	-0.132
	Control / 25	18	2	98.8	0.917	0.734	-0.350	20	2	96.6	0.851	0.683	-0.259
	Control / 250	18	1	99.1	0.919	0.209	-0.259	19	3	99.6	0.926	0.756	-0.450
PND 50	5 treatment groups	48	3	69.7	0.644	0.121	-0.297	47	3	80.0	0.504	0.346	-0.452
	Control / 0.25	19	2	99.9	0.994	0.271	-0.349	20	3	99.4	0.933	0.566	-0.444
	Control / 2.5	20	2	99.4	0.972	0.334	-0.326	20	3	99.3	0.939	0.571	-0.432
	Control / 25	20	2	99.6	0.983	0.309	-0.311	18	1	89.8	0.762	0.253	-0.210
	Control / 250	20	2	99.6	0.988	0.305	-0.358	19	4	99.3	0.896	0.663	-0.484
PND 90	5 treatment groups	48	2	47.0	0.449	0.039	-0.167	52	3	63.0	0.517	0.239	-0.321
	Control / 0.25	20	2	97.8	0.797	0.588	-0.234	21	1	97.5	0.893	0.110	-0.208
	Control / 2.5	19	2	99.8	0.998	0.060	-0.339	20	1	99.4	0.977	0.192	-0.219
	Control / 25	20	2	99.6	0.993	0.142	-0.264	20	2	99.5	0.942	0.329	-0.335
	Control / 250	19	3	99.4	0.884	0.807	-0.362	20	3	96.3	0.667	0.830	-0.326
PND 140	5 treatment groups	49	2	45.7	0.429	0.022	-0.179	41	3	58.2	0.357	0.244	-0.333
	Control / 0.25	19	3	99.7	0.950	0.815	-0.200	17	2	79.5	0.548	0.422	-0.238
	Control / 2.5	20	3	98.8	0.938	0.755	-0.264	17	3	99.4	0.961	0.578	-0.444
	Control / 25	19	2	99.8	0.997	0.344	-0.296	18	2	99.0	0.919	0.600	-0.367
	Control / 250	20	2	94.9	0.763	0.603	-0.246	19	3	99.6	0.968	0.493	-0.504
PND 200	5 treatment groups	50	2	42.5	0.378	0.022	-0.197	49	2	43.4	0.396	0.051	-0.209
	Control / 0.25	20	4	98.8	0.896	0.750	-0.392	20	3	97.5	0.807	0.514	-0.397
	Control / 2.5	20	2	93.7	0.670	0.646	-0.178	19	2	97.7	0.888	0.372	-0.309
	Control / 25	20	3	99.8	0.989	0.454	-0.467	19	3	98.9	0.913	0.487	-0.480
	Control / 250	21	2	98.8	0.817	0.633	-0.322	19	2	92.2	0.633	0.415	-0.281

^a^ number of observations in the dataset

^*b*^ number of latent variables included in the PLS-DA model

^*c*^ % of variance explained by the PLS-DA model

^*d*^ predictive ability of the PLS-DA model calculated by 7-fold cross-validation

^*e*^ Intercept values (R2/Q2) of permutation test. The intercept values (R2/Q2) represent the values of R2 and Q2 of a purely random model. A robust model has a Q^2^ intercept value < 0.

### A-SCA analyses, serum samples

We further explored the means to overcome the limits of PLS-DA for single time point / multiple dose comparisons by using A-SCA. The A-SCA method, which works in two stages (ANOVA and PCA), was applied to the entire dataset (5 time points and 5 doses). The first step of A-SCA allowed us to determine that the overall variability in serum samples was split among the experimental factors, with contributions of 23.6%, 0.9% and 4.6%, for the Time factor, the Dose factor, and the Time*Dose interaction, respectively. Time was the only significantly altered factor (p-value = 0.0001). Residuals contributed to a major part of the variability (69.9%). In the second step of A-SCA, we focused on the Time factor. In this context, the first principal component (PC) of PCA explained 61.4% of the mean dynamic variation. Twenty-six metabolites were identified as significantly different between these five time-points (S2 Table).

**Fig 5:**
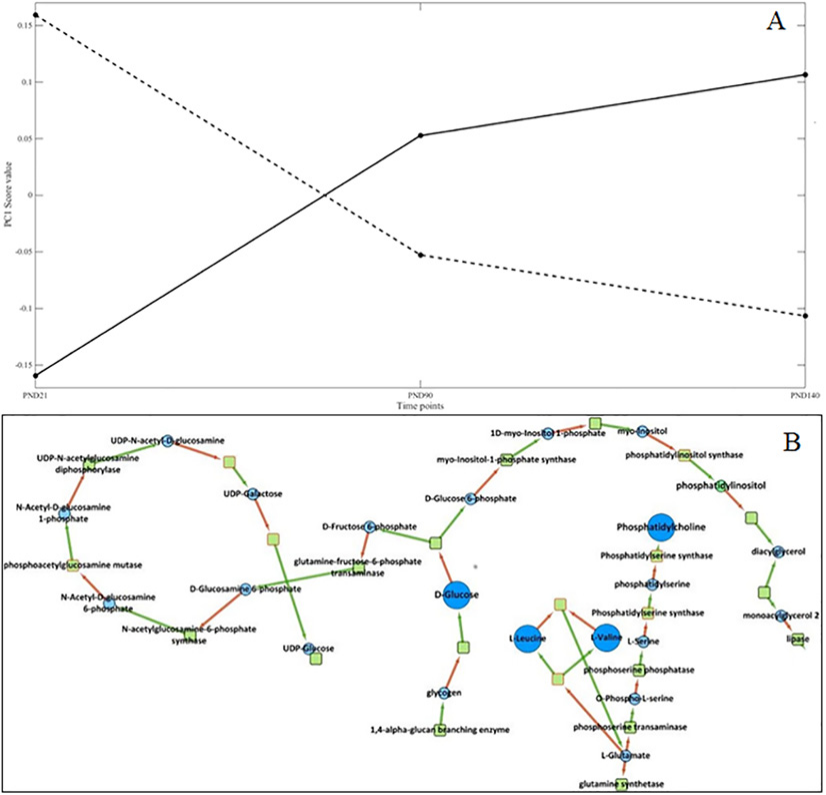
A-SCA of serum samples ("Reduced" dataset). A) One-dimensional PCA score plot of the Time * Dose interaction. The horizontal axis represents the time course, and the vertical axis represents the score value for the first principal component (86.1% of explained variability). Each dot represents the average of animals euthanized at the time point mentioned on the horizontal axis. The solid line corresponds to BPA0.25 and the dashed line corresponds to BPA25. B) Metabolic pathways modulated by concentration (0.25 *Vs* 25μg /kg BW/day) over time focusing on the biochemical pathways including the metabolites in which concentrations were found to be increased.

We further took advantage of the A-SCA method to identify Time*Dose significant interactions. To this end, we examined all combinations of *k* time points and *h* treatment groups. Among these combinations, A-SCA enabled us to identify a significant Time*Dose interaction for the PND21, PND90 and PND140 groups, when exposed perinatally to either 0.25μg or 25μg BPA/kg BW/day. For this "reduced" dataset, the Time factor represented 20.0% of the total variation (p-value<0.0001), the Dose factor 0.9% and the Time*Dose interaction 7.3% (p-value = 0.0385). For the Time sub-model, the first PC explained 86.6% of the dynamic mean variation. The corresponding list of discriminant metabolites (provided in S3 Table) shows that the metabolism of amino-acids, glucose and fatty acids was modified along the time course. For the Time*Dose sub-model, the first PC explained 86.1% of the variation corresponding to the interaction of the two factors. The score profile of the first principal component of the Time * Dose interaction describes the different responses over time for both the BPA0.25 and BPA25 doses (Fig 5A). For these, the unequivocal difference in the score profiles clearly demonstrates distinct effects of BPA on the metabolome over time according to the dose, also suggesting different mechanisms of action that will have to be further investigated. The concentrations of betaine, choline, citrate, glucose, glutamate, glycine, histidine, isoleucine, lactate, leucine and lipids were modified (S4 Table). We further analyzed this Time*Dose interaction by modeling the metabolic network and extracting the sub-networks in which our discriminant metabolites were involved. The reconstruction of the metabolic network and the study of its modulation after BPA perinatal exposure (0.25μg vs 25μg) (Fig 5B) highlighted a modulation of the myo-inositol pathway over time, linking at least 2 discriminant metabolites, namely glucose and glycerol. In addition, leucine and valine were also involved in the modulation of the phosphatidylcholine pathway.

### A-SCA analysis, liver samples

When applied to the entire dataset, A-SCA first led us to determine that the variability in liver extract samples was associated with the Time factor (46.2% of the variability), the Dose factor (2.5%) and the Time*Dose interaction. Both the Time and Dose factors were significant (p = 0.001), and the residual variability (48.5%) was lower than that for serum. For the Time sub-model, 89.2% of the average dynamic variation was explained by a single PC. Concentrations of alanine, glucose, glycine, lactate and lysine was higher in PND21 extracts, compared to PND50, PND90, PND140 and PND200, respectively. For betaine, creatine, glutamate, glutathione, oxidized glutathione, glycogen, inosine and valine, it was the opposite (S2 Table). For the Dose sub-model, the first PC explained 88.1% of the dose-specific variation. The concentrations of choline, hypotaurine and phosphocholine were lower in the Control group compared to all BPA groups. Regarding the liver, A-SCA did not unveil a significant combination for the Time*Dose interaction.

## Discussion

Over the last decade, the low dose effects of endocrine disruptors have been a focus of research [3, 5]. Perinatal exposure to xeno-estrogens such as BPA resulted in lasting changes at the level of the genital tract and the reproductive function of mice [4, 27], as well as in metabolic disorders [12, 28, 29]. These latter effects were uncovered only recently, and their underlying mechanisms are still unknown. The recent development of metabolomics has opened new perspectives in the field of low dose toxicology. Using ^1^H-NMR metabolomics, we have now shown that exposing pregnant mice to BPA, even at very low doses, results in significant metabolic shifts in pups measured soon after birth (PND2) [13]. Moreover, the current metabolomic study showed that a significant discrimination between exposed and non-exposed pups could be demonstrated in the tissues (liver, blood, brain), at PND21.

Here, we also performed a similar type of metabolomic analyses using tissues collected from SD rats born from mothers exposed during pregnancy and early lactation to different doses of BPA ranging from 0.25 to 250 μg/kg BW/d. The study was designed to identify long lasting metabolic effects, and to examine whether BPA effects could be detected up to 6 months post exposure, despite the major physiological metabolic changes which are occurring throughout the individual’s lifetime (puberty, aging). It was previously reported that perinatal exposure to BPA results in altered behaviors, attenuation of sexually dimorphic characters, obesity, altered estrus cyclicity, altered LH regulation and mammary cancer [30–32]. Our current goals were to examine whether long lasting effects of BPA could be demonstrated through a metabolomic approach and to develop the statistical approaches which would allow us to examine combined time and dose effects for large matrices of data involving separate groups of animals. The latter issue is of great concern in the field of long lasting effects explored through metabolomics, since the variability associated with aging adds to the variability associated with the treatment. With this purpose in mind, both PLS-DA and A-SCA were used based on a 5*5 matrix (5 doses: controls + 4 different BPA doses; 5 time points).

PLS-DA is the current method of choice in metabolomics seeking discrimination between samples originating from exposed/unexposed animals. Only recently has it been applied to the field of endocrine disruption [13, 33]. In CD1 mice, liver extract analyses at PND21 demonstrated changes connected with a modulation of energy metabolism [13]. In the present study, we confirmed that PLS-DA is a reliable method for metabolomic studies related to EDC, and that perinatal BPA exposure produces significant metabolic changes in liver and serum composition. All single time point entire set PLS-DA models (serum and liver) were valid, as well as all pairwise models, with the only exception being the BPA2.5 dose at PND21 in serum. This large scale study extends our previous results beyond PND21, and shows that BPA modulates the expression of the metabolome of animals exposed perinatally. Even at PND200, metabolomic biomarkers can still discriminate all groups of animals from controls, and all doses from each other. As in mice, several pathways related to energy metabolism in rats were found to be involved in the discrimination between groups. These results unequivocally indicate significant shifts of the metabolome, due to BPA exposure. Moreover, these changes are consistent with previous reports based on targeted biochemical measurements [28, 34]. It should be also emphasized that BPA-associated changes in liver metabolism, whether resulting from direct or indirect mechanisms, were always found to be concomitant with changes observed in serum samples. Since serum samples can be obtained by non-invasive procedures, this suggests that similar studies might be conducted in human beings with the aim of examining the effects of xeno-estrogens, could be carried out based on blood sampling, with reasonable chances of success, unless the variability associated with genetic differences and/or with effects of chemicals mixture hinders the discriminative power of metabolomics.

When applied to each time point separately, PLS-DA analysis successfully discriminated between the control group and all BPA-exposed groups, but did not allow a 5 Doses*5 Time-points exploration of the data. The corresponding models, both for the serum and liver, were neither valid nor robust. These analyses of the full set of data (e.g. 25 groups) clearly suggested, instead, that a main reason for the non-validity of these models was due to the contribution of the variability associated with age, especially around PND21 (whatever the dose), which masked dose-related differences. This was unsurprising since major metabolic changes occur around PND21 in connection with the onset of puberty. It is therefore to be expected that when analyzing a whole data set, low-dose effects of EDC could become "masked" in comparable long-term studies by physiological changes and aging. We explored means to overcome such difficulty by using A-SCA. A-SCA, unlike PLS-DA, can take into account both data’s temporal and multivariate structure. A-SCA highlighted dynamic variations of the metabolome (serum and liver extracts), BPA dose related effects (liver extracts) and Time*Dose interaction effects (on selected sets of time-points and doses, for serum samples).

A-SCA has only been used in a few studies to assess metabolic changes connected with Time*Dose using a nested design [14, 35]. In those studies, like in ours, a large part of the variation could not be explained by the experimental factors. Residual variation included experimental and individual variability. Despite this difficulty, robust models could be constructed in our study combining A-SCA with permutation tests. In the liver, not only could BPA effects could still be detected at PND200, but A-SCA demonstrated that the Dose factor was still detectable and significant (2.5% of the variability) when taking the entire model (5 doses, 5 time points) into account. This means that the liver metabolome is significantly modulated by BPA perinatal imprinting, even when taking into account the variability linked with maturation and aging. Regarding serum, BPA's effects taken alone were not significant in the A-SCA model, but the Time*Dose interaction was clearly evident for a sub-model included the PND21, PND90 and PND140 groups.

As highlighted by the score profiles, both for the liver and serum, and in good agreement with the results of PLS-DA, major changes occurred around puberty (up to PND50) which could not be obvious when analyzing all time-points together. Serum samples provide a "picture" of the circulating endogenous metabolites. For the liver, we observed both time and dose effects, suggesting that the impact of BPA on the hepatic function could be a lasting one, or more pronounced, over time. Moreover, the variability explained by the Time factor was greatly increased when PND21 was included in the A-SCA model.

Additionally, by extracting sub-networks from the whole network of reactions connecting the different biomarkers, we were able to visualize the metabolic biochemical cascades of reactions that were altered by BPA. The myo-inositol pathway was found to be modulated for the BPA0.25/PND21 and BPA25/PND90 interactions, respectively. Myo-inositol plays an important role in several biological processes, including insulin signal transduction, cytoskeleton assembly, nerve guidance (epsin), intracellular calcium concentration control, cell membrane potential maintenance, breakdown of fats, and reduction of blood cholesterol. Leucine and valine are linked to the phosphatidylcholine modulation pathway. Phosphatidylcholine is a major constituent of cell membranes and of pulmonary surfactant, and plays a role in membrane-mediated cell “signaling”. In the liver, the respective concentrations of choline, phosphocholine and hypotaurine were lower in the Control group, compared to all BPA groups. Choline, as the precursor molecule for the neurotransmitter acetylcholine is involved in multiple functions including memory and muscle control. Choline and its metabolites play an important role on the structural integrity of the cell (membranes) as well as cholinergic neurotransmission (acetylcholine synthesis). Through its metabolite betaine, choline is also a major source for methyl groups, which participate in the S-adenosylmethionine synthesis pathways. On these bases, it appears that BPA may modify the cell membrane constitution, possibly interfering with “signaling” pathways. BPA may also modulate the secretion of the endogenous neurotransmitter hypotaurine, which acts at the level of glycine receptors.

In conclusion, PLS-DA highlighted unequivocal metabolic shifts in sera and the liver of rats perinatally exposed to low doses of BPA. By applying A-SCA, the further separation of the total variation according to the different experimental factors was accomplished, which in turn can be analyzed independently. A-SCA enabled the observation of a dynamic, time/age dependent evolution of serum metabolome throughout the rat’s life span; these data could not have been provided by PLS-DA modeling alone. For low doses of BPA, a specific time course modulation of the serum metabolome was found to be associated with a Dose*Time interaction. For the liver, BPA effects were found to be significant even when the whole data set (5 different time points, 5 BPA doses) was analyzed. The variables responsible for the discrimination between groups strongly indicate that this model xeno-estrogen, i.e., BPA, is able to modulate energy metabolism. In addition, several disrupted metabolic pathways hint at mechanisms related to a potential disruption of signaling pathways and neuro-transmitters, which may well be linked with the BPA-induced neuro-developmental defects already demonstrated in rodents.

## Supporting Information

S1 Fig Experimental design for each BPA dose (h = Control, BPA0.25μg, BPA2.5μg, BPA25μg or BPA250μg). n_hk_ represents the number of observations for each BPA dose (h) and time point (k).
(TIF)

S2 Fig Two-dimensional PLS-DA scores plot of integrated ^1^H-NMR spectra (whole dataset) A. Serum samples: 2 components, R^2^ = 6.8% and Q^2^ = 0.059. B. Liver extracts: 2 components, R^2^ = 7.5% and Q^2^ = 0.053. (TIF)

S1 Table ^1^H and ^13^C resonance assignments with chemical shifts, multiplicity and J-couplings for signals identified in rat serum and liver samples. (DOCX)

S2 Table Discriminant metabolites identified for the Time sub-model in A-SCA for *Serum Samples (S)* and Liver Extracts (L) ("-": decrease in the mean concentration; "+": increase in the mean concentration). (DOCX)

S3 Table Discriminant metabolites identified for serum samples for the Time sub-model in A-SCA ("Reduced" Dataset) ("-": decrease in the mean concentration; "+": increase in the mean concentration). (DOCX)

S4 Table Discriminant metabolites identified for serum samples for the Interaction Time*Dose sub-model in A-SCA; "reduced" dataset; ("-": decrease in the mean concentration; "+": increase in the mean concentration). (DOCX)

S5 Table Normalized Bucket NMR Data of Serum Samples. (XLSX)

S6 Table Normalized Bucket NMR Data of Aqueous Liver Extracts. (XLSX)

S1 Text Supplementary information about the A-SCA method and its impletmentation in the current study. (DOCX)
