# Supplementary figures and images for: Dynamic Metabolic Disruption in Rats Perinatally Exposed to Low Doses of Bisphenol-A
Source: PLoS One. 2015 Oct 30;10(10):e0141698. doi: 10.1371/journal.pone.0141698 (PMC4627775; doi:10.1371/journal.pone.0141698)

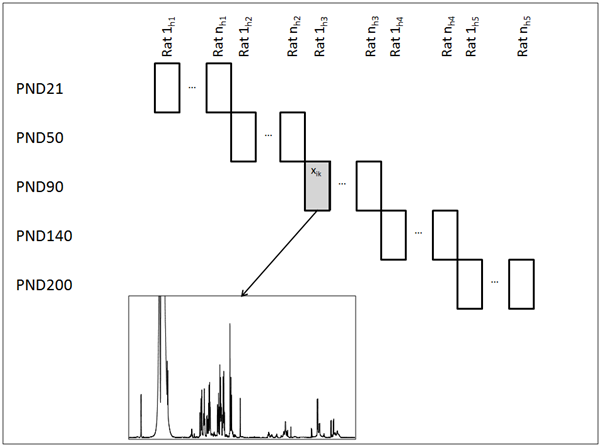

Supplement: S1 Fig — nhk represents the number of observations for each BPA dose (h) and time point (k). (TIF) [file pone.0141698.s001.tif]

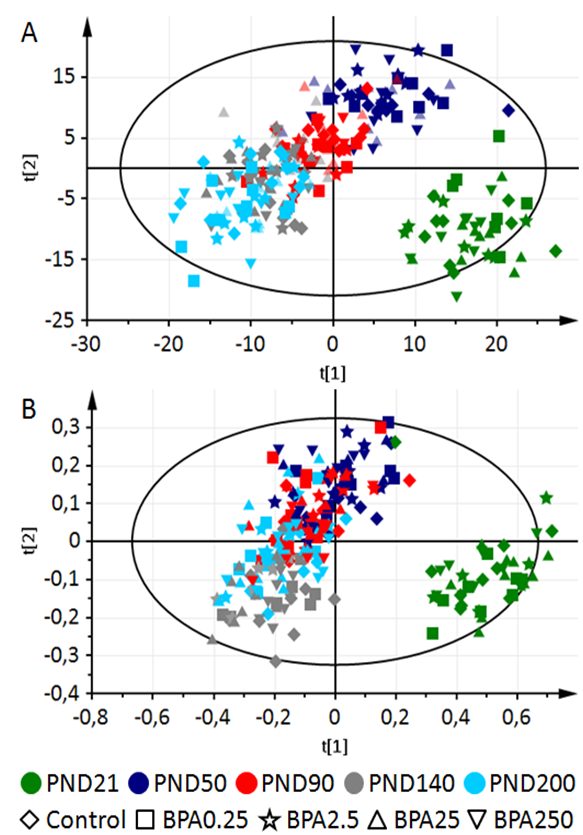

Supplement: S2 Fig — (TIF) [file pone.0141698.s002.tif]
