# Supplementary material for: Dynamic Metabolic Disruption in Rats Perinatally Exposed to Low Doses of Bisphenol-A
Source: PLoS One. 2015 Oct 30;10(10):e0141698. doi: 10.1371/journal.pone.0141698 (PMC4627775; doi:10.1371/journal.pone.0141698)
Supplement: S1 Table — (DOCX) [file pone.0141698.s003.docx]

**Table S1.**^1^H and ^13^C resonance assignments with chemical shifts, multiplicity and J-couplings for signals identified in rat serum and liver samples.

| Compound | 1H ppm (multiplicity, coupling constant, assignment)/13C ppm | Biological matrix |
| --- | --- | --- |
| Acetate (ChEBI 30089) | 1.92 (s, CH_3_)/25.9 | Plasma, liver |
| Alanine (ChEBI 16977) | 1.48 (d, J=7.1 Hz, CH_3_)/18.9 ; 3.78 (q, J=7.2 Hz, CH)/53.1 | Plasma, liver |
| Betaine | 3.27(s, CH_3_)/56.1 ; 3.91(s, CH_2_) /68.9 | Plasma, liver |
| Choline (ChEBI 15364) | 3.22(s, N(CH_3_)_3_)/56.9 ; 3.52(m, NCH_2_)/70.0 ;  4.05(m, CH_2_)/58.3 | Plasma, liver |
| Citrate (ChEBI 50744) | 2.54(d, J=15 Hz, 2CH)/48.7 ; 2.70(d, J=15 Hz, 2CH)/48.7 | Plasma |
| Creatine (ChEBI 16919) | 3.93(s, CH_2_)/56.4 ; 3.03(s, CH_3_)/39.9 | Plasma, liver |
| Creatinine (ChEBI 16737) | 4.05(s, CH_2_)/59.1 ; 3.04(s, CH_3_)/33.1 | Plasma |
| Formate (ChEBI 15740) | 8.45(s,CH) | Plasma, liver |
| Fumarate (ChEBI 29806) | 6.53(s,CH) | Liver |
| α-Glucose (ChEBI 17634) | 3.41(m, CH)/72.3; 3.54(m, CH)/73.9; 3.71(m, CH)/75.4; 3.83(m, CH)/74.2; 3.84(m, CH)/63.3; 5.23 (d, CH, J=3.8 Hz)/94.8 | Plasma, liver |
| β-Glucose (ChEBI 17634) | 3.25(m, CH)/76.9; 3.41(m, CH)/72.3; 3.48(m, CH)/78.4; 3.73(m, CH)/63.3; 3.90(m, CH)/63.4; 4.64 (d, CH, J=8 Hz)/98.6 | Plasma, liver |
| Glutamate (ChEBI 29988) | 2.06 (m, CH_2_)/29.6; 2.36 (m, CH_2_)/36.1; 3.78(m, CH)/56.7 | Plasma, liver |
| Glutamine (ChEBI 18050) | 2.16(m, CH_2_)/29.1; 2.46(m, CH_2_)/33.6 ; 3.78(m, CH)/56.7 | Plasma, liver |
| Glutathione (oxidized)  (ChEBI 17858) | 2.18(m, CH_2_)/29.1; 2.55(m, CH_2_)/34.2; 2.98(m, CH)/28.3; 3.31(m, CH)/41.6 ; 3.78(m, CH_2_)/46.2; 3.78(m, CH)/56.7 | Liver |
| Glutathione (reduced)  (ChEBI 16856) | 2.18(m, CH_2_)/29.1; 2.55(m, CH_2_)/34.2; 2.98(m, CH_2_)/28.3; 3.78(m, CH_2_)/46.2; 3.78(m, CH)/56.7; 4.56(m,CH)/58.5 | Liver |
| Glycine (ChEBI 15428) | 3.56(s, CH_2_)/44.3 | Plasma, liver |
| Glycogen (ChEBI 28087) | 3.66(m, CH)/79.3; 3.98 (m, CH)/75.8; 5.41(m, CH)/102.2 | Liver |
| Hypotaurine (ChEBI 16668) | 2.65(t,J=7Hz,CH_2_)/57.9; 3.36(t,J=7Hz,CH_2_)/35.9 | Liver |
| Inosine (ChEBI 17596) | 4.28(m, CH)/88.2; 4.44(m, CH)/73.1; 6.11(d, J=5.7 Hz, CH ring)/90.9 ; 8.23(s, CH ring)/148.9; 8.32 (s, CH ring)/ 142.7 | Liver |
| Isoleucine (ChEBI 17191) | 0.93(t, J=7.2 Hz, CH_3_)/13.9 ; 0.99 (d, J=7.2 Hz, CH_3_)/17.4 | Plasma, liver |
| Lactate (ChEBI 24996) | 1.33(d, J=7.2 Hz, CH_3_)/22.9 ; 4.11(q, J=7.2 Hz, CH)/71.2 | Plasma, liver |
| Leucine (ChEBI 15603) | 0.94(d, J=6 Hz, CH_3_)/23.6; 0.97(d, J=6 Hz, CH_3_)/24.7 ;  1.71(m, CH)/27.3; 1.71(m, CH_2_)/42.6 | Plasma, liver |
| Non-saturated Lipids | 2.70(m, CH_2_); 5.30(m, CH=CH)/132.4 | Plasma |
| Lipids (LDL, VLDL) | 0.87(m, CH_3_)/14.7 ; 1.28(m, CH_2_)/34.6; 1.59(m,CH_2_)/27.7 | Plasma |
| Lysine (ChEBI 18019) | 1.48(m, CH_2_)/24.6 ; 1.72(m, CH_2_)/29.1; 1.91(m, CH_2_)/32.7 ; 3.01(m, CH_2_)/42.1 | Plasma, liver |
| Nicotinic acid (ChEBI 15940) | 7.60 (dd, J=7.9 and 5 Hz, CH ring); 8.25(m, CH ring) ;  8.58(m, CH ring) ; 8.92(s, CH ring) | Liver |
| Phenylalanine (ChEBI 17925) | 7.43(t, J=7 Hz, CH ring) ; 7.38(t, J=7 Hz, CH ring) ;  7.32 (d, J=7 Hz, CH ring) | Plasma, liver |
| Phosphorylcholine (ChEBI 18132) | 3.22(s, N(CH_3_)_3_)/56.9 ; 3.60(m, CH_2_)/69.4 ; 4.18(m, CH_2_)/60.7 | Plasma, liver |
| Proline (ChEBI 17203) | 2.0(m,CH_2_)/26.5; 2.06(m,CH_2_)/31.8; 2.36(m,CH_2_)/31.7; 3.34(m,CH_2_)/48.8; 3.42(m,CH_2_)/48.8; 4.13(m,CH)/63.9 | Plasma |
| Pyruvate (ChEBI 15361) | 2.38 (s, CH_3_) / 29.1 | Plasma |
| Succinate (ChEBI 30031) | 2.41 (s, CH_2_)/36.8 | Liver |
| Taurine (ChEBI 15891) | 3.26(t, J=7.3 Hz, CH_2_)/49.9 ; 3.43(t, J=7.3 Hz, CH_2_)/38.1 | Plasma, liver |
| Threonine (ChEBI 16857) | 1.32(d, J=7Hz, CH3)/21.9 ; 3.59(m,CH)/63.2 ; 4.25(m,CH)/68.7 | Plasma, liver |
| Tyrosine (ChEBI 17895) | 6.90 (m, CH ring); 7.18(m, CH ring) | Plasma, liver |
| Uridine (ChEBI 16704) | 4.14(m, CH)/86.9 ; 4.38(m, CH)/76.4 ; 5.88(m, CH ring)/104.9 ; 5.90 (m, CH)/92.1 ; 7.88 (d, J=8.10 Hz, CH ring)/144.5 | Liver |
| Valine (ChEBI 16414 | 0.99 (d, J=, CH_3_)/19.5; 1.05(d, J=, CH_3_)/20.7; 2.28(m,CH)/31.9 | Plasma, liver |
