# Supplementary material for: Dynamic Metabolic Disruption in Rats Perinatally Exposed to Low Doses of Bisphenol-A
Source: PLoS One. 2015 Oct 30;10(10):e0141698. doi: 10.1371/journal.pone.0141698 (PMC4627775; doi:10.1371/journal.pone.0141698)
Supplement: S2 Table — (DOCX) [file pone.0141698.s004.docx]

**Table S2.** Discriminant metabolites identified for the Time sub-model in A-SCA for *Serum Samples (S)* and **Liver Extracts (L)** ("-": decrease in the mean concentration; "+": increase in the mean concentration).

| Metabolites | PND21/ | | PND21/ | | PND21/ | | PND21/ | | PND50/ | | PND50/ | | PND50/ | | PND90/ | | PND90/ | | PND140/ | |
| --- | --- | --- | --- | --- | --- | --- | --- | --- | --- | --- | --- | --- | --- | --- | --- | --- | --- | --- | --- | --- |
|  | PND50 | | PND90 | | PND140 | | PND200 | | PND90 | | PND140 | | PND200 | | PND140 | | PND200 | | PND200 | |
|  | *S* | **L** | *S* | **L** | *S* | **L** | *S* | **L** | *S* | **L** | *S* | **L** | *S* | **L** | *S* | **L** | *S* | **L** | *S* | **L** |
| Acetate |  | + |  | + |  | + | - | + |  |  |  |  |  |  |  |  |  |  |  |  |
| Alanine |  | + | - | + | - | + | - | + | - |  | - |  | - | + | - |  |  |  |  |  |
| Betaine | - | - | - | - | - | - | - | - | - | - | - |  | - | - |  |  |  |  |  |  |
| Choline | - | + | - | + | - | + | - | + | - |  | - | - | - | - | - |  | - |  |  |  |
| Citrate | - |  | - |  | - |  | - |  | - |  | - |  | - |  |  |  |  |  |  |  |
| Creatine |  |  |  |  |  |  |  |  |  |  |  |  | - |  |  |  |  |  |  |  |
| Ethanolamine | - |  | - |  | - |  | - |  | - |  | - |  | - |  | - |  | - |  |  |  |
| Dimethylglycine | - |  | - |  | - |  | - |  | - |  | - |  | - |  | - |  | - |  |  |  |
| Glucose | + | + | + | + | + | + | - | + | - | + | - | + | - | + |  | + | - |  |  | - |
| Glutamate | - | - | - | - | - | - | - | - |  | - | - | - | - | - | - | - | - |  |  | - |
| Glutamine | - | - |  | - | - | - | - | - |  |  | - | - | - |  | - |  | - |  |  |  |
| Glutathione |  | - |  | - |  | - |  | - |  | - |  | - |  | - |  | - |  | - |  | + |
| Glycerol |  |  | + |  | + |  | + |  |  |  | + |  | + |  |  |  |  |  |  |  |
| GPC*^a^* |  |  | - |  | - |  | - |  | - |  | - |  | - |  |  |  |  |  |  |  |
| Glycine |  | + | - | + | - | + | - | + | - |  | - |  | - |  |  |  |  |  |  |  |
| Glycogen |  | - |  | - |  | - |  | - |  |  |  | - |  | - |  | - |  | - |  |  |
| Hypotaurine |  | + |  | + |  | + |  | + |  |  |  | - |  | - |  |  |  |  |  |  |
| Inosine |  |  |  | - |  | - |  | - |  |  |  | - |  | - |  |  |  |  |  |  |
| Lactate | - | + | - | + | - | + | - | + | + | + | - | + | - | + | - | + | - | + |  |  |
| Leucine |  |  |  |  |  |  | + |  |  |  |  |  |  |  |  |  |  |  |  |  |
| Lipids | - |  | + |  | + |  | + |  | + |  | + |  | + |  | + |  | + |  |  |  |
| Unsaturated lipids | - |  | + |  | + |  | + |  | + |  | + |  | + |  | + |  | + |  |  |  |
| Lysine | - | + | - | + | - | + | - | + | - |  | - | - | - |  | - | - | - |  | - |  |
| Methionine | - |  | - |  | - |  | - |  |  |  | - |  | - |  | - |  | - |  |  |  |
| Phosphorylcholine | - | + | - | + | - | + | - | + |  |  | - |  | - |  |  |  |  |  |  |  |
| Proline | - |  | - |  | - |  | - |  | - |  | - |  | - |  | - |  | - |  |  |  |
| Pyruvate | - |  | - |  | - |  | - |  |  |  | - |  | - |  | - |  | - |  |  |  |
| Serine | - |  | - |  | - |  | - |  | - |  | - |  | - |  | - |  | - |  |  |  |
| Succinate |  |  |  | + |  |  |  | + |  | + |  | + |  | + |  | + |  | + |  |  |
| Tyrosine | - |  | - |  | - |  | - |  |  |  | - |  | - |  | - |  | - |  |  |  |
| Valine | + |  | + | - |  |  | + | - |  | - | - | - | - | - | - | - |  |  |  | + |

*^a^* Glycerophosphocholine
