# Supplementary material for: Dynamic Metabolic Disruption in Rats Perinatally Exposed to Low Doses of Bisphenol-A
Source: PLoS One. 2015 Oct 30;10(10):e0141698. doi: 10.1371/journal.pone.0141698 (PMC4627775; doi:10.1371/journal.pone.0141698)
Supplement: S3 Table — (DOCX) [file pone.0141698.s005.docx]

**Table S3.** Discriminant metabolites identified for serum samples for the Time sub-model in A-SCA ("Reduced" Dataset) ("-": decrease in the mean concentration; "+": increase in the mean concentration).

| Metabolites | P-Value^1^ | PND21/PND90 | PND21/PND140 | PND90/PND140 |
| --- | --- | --- | --- | --- |
| Alanine | 4,96E-05 |  | - | - |
| Betaine | 2,30E-08 | - | - |  |
| Choline | 2,49E-06 | - | - | - |
| Citrate | 9,23E-09 | - | - | - |
| Creatine | 4,46E-06 | - | - |  |
| Ethanolamine | 2,46E-09 | - | - | - |
| Formate | 4,18E-08 | - | - |  |
| Glucose | 5,88E-06 | + | + | - |
| Glutamate | 3,51E-06 | - | - | - |
| Glutamine | 8,73E-08 | - | - |  |
| Glycerol | 2,46E-04 | + | + |  |
| Glycerophosphocholine | 1,58E-02 | - | - |  |
| Glycine | 4,27E-08 | - | - |  |
| Histidine | 7,47E-09 | - | - |  |
| Isoleucine | 9,90E-04 | + |  |  |
| Leucine | 7,43E-04 | + |  | + |
| Lipids | 2,97E-08 | + | + | - |
| Lysine | 2,31E-08 | - | - |  |
| Methionine | 8,19E-09 | - | - | - |
| Phenylalanine | 2,49E-07 | - | - |  |
| Phosphocholine | 9,14E-08 | - | - |  |
| Proline | 2,85E-08 | - | - | - |
| Pyruvate | 6,65E-07 |  | - | - |
| Serine | 5,59E-06 | - | - | - |
| Taurine | 2,07E-02 |  | - |  |
| Threonine | 7,69E-07 | + | + | - |
| Tyrosine | 7,47E-09 | - | - | - |
| Valine | 7,28E-06 | + | + | - |

^1^Kruskal-Wallis test.
