# Supplementary material for: Dynamic Metabolic Disruption in Rats Perinatally Exposed to Low Doses of Bisphenol-A
Source: PLoS One. 2015 Oct 30;10(10):e0141698. doi: 10.1371/journal.pone.0141698 (PMC4627775; doi:10.1371/journal.pone.0141698)
Supplement: S4 Table — (DOCX) [file pone.0141698.s006.docx]

**Table S4.** Discriminant metabolites identified for serum samples for the Interaction Time*Dose sub-model in A-SCA; "reduced" dataset; ("-": decrease in the mean concentration; "+": increase in the mean concentration).

| Metabolites | 0.25*^a^*_21*^b^*/ 0.25_90 | 0.25_21/ 0.25_140 | 0.25_21/ 25_21 | 0.25_21/ 25_90 | 0.25_21/ 25_140 | 0.25_90/ 0.25_140 | 0.25_90/ 25_21 | 0.25_90/ 25_90 | 0.25_90/ 25_140 | 0.25_140/ 25_21 | 0.25_140/ 25_90 | 25_21/ 25_90 | 25_21/  25_140 | 25_90/ 25_140 |
| --- | --- | --- | --- | --- | --- | --- | --- | --- | --- | --- | --- | --- | --- | --- |
| Alanine |  | - |  |  |  |  |  |  |  | + |  |  | - |  |
| Betaine | - | - |  | - | - |  | + |  |  | + |  | - | - |  |
| Choline |  | - |  |  | - |  |  |  |  | + |  |  | - |  |
| Citrate | - | - |  | - | - |  | + |  |  | + |  | - | - |  |
| Ethanolamine | - | - |  | - | - |  | + |  |  | + |  |  | - |  |
| Glucose | - | - | - | + | + |  |  |  |  | - |  | + | + |  |
| Glutamate |  | - |  |  | - |  |  |  |  | + | + | - | - | - |
| Glutamine |  | - |  |  |  |  |  |  |  | + |  |  |  |  |
| Glycerol |  |  |  | + | + |  |  |  |  |  | + |  | + |  |
| GPC*^c^* | - | - |  |  |  |  |  |  |  |  |  |  |  |  |
| Glycine | - | - |  |  | - |  | + |  |  | + |  |  | - |  |
| Isoleucine | + |  |  |  |  | - |  |  | - |  |  |  |  |  |
| Leucine | + |  |  | + |  |  |  |  | - |  |  |  |  |  |
| Lipids | + | + |  | + | + |  | + |  |  | - |  | + | + |  |
| Lysine | - | - |  |  | - |  | + |  |  | + |  |  | - |  |
| Phosphocholine | - | - |  | - | - |  | + |  |  | + |  | - | - |  |
| Proline |  | - |  |  | - |  | + |  |  | + |  | - | - |  |
| Pyruvate |  | - |  |  | - |  |  |  |  | + |  |  | - |  |
| Serine |  | - |  |  | - |  |  |  |  | + | + |  | - | - |
| Succinate | - | - |  | - | - |  | + |  |  | + |  | - | - |  |
| Threonine | + |  |  | + | + |  | - |  |  |  |  | + | + |  |
| Tyrosine | - | - |  | - | - |  | + |  |  | + |  | - | - |  |
| Valine | + |  |  | + |  |  | - |  |  |  |  | + |  |  |

*^a^* BPA Dose (BPA0.25/BPA25)

*^b^* Time-point (PND21/PND90/PND140)

*^c^* Glycerophosphocholine
