# Supplementary material for: Dynamic Metabolic Disruption in Rats Perinatally Exposed to Low Doses of Bisphenol-A
Source: PLoS One. 2015 Oct 30;10(10):e0141698. doi: 10.1371/journal.pone.0141698 (PMC4627775; doi:10.1371/journal.pone.0141698)
Supplement: S1 Text — (DOCX) [file pone.0141698.s009.docx]

**Dynamic metabolic disruption in rats perinatally exposed to low doses of bisphenol-A**

Marie Tremblay-Franco^1^, Nicolas J. Cabaton^1^, Cécile Canlet^1^, Roselyne Gautier^1^, Cheryl M. Schaeberle^2^, Fabien Jourdan^1^, Carlos Sonnenschein^2^, Florence Vinson^1^, Ana M. Soto^2^, and Daniel Zalko^1*^

*^1^* Institut National de la Recherche Agronomique (INRA), UMR1331, TOXALIM (Research Centre in Food Toxicology), Université de Toulouse, Toulouse, France

*^2^* Department of Integrative Physiology & Pathobiology, Tufts University School of Medicine, Boston, Massachusetts, USA.

*^*^* Corresponding author:

E-mail: daniel.zalko@toulouse.inra.fr (DZ)

**Material and methods**

The A-SCA method allows for the separation of total variation into parts due to each experimental factor. Effects of each factor can therefore be analyzed independently. A-SCA combines Analysis of Variance (ANOVA) and Principal Component Analysis (PCA).

In the first step of A-SCA, **ANOVA** is used to split the total variation (Equation 1):

x_hkij_ = μ_j_ + α_kj_ + β_hj_ + (αβ)_khj_ + ε_hkij_,

where *x_hkij_* represents the value of the NMR bucket *j* (j=1, ...,J) measured on the individual *i* dosed with treatment *h* (h=Control, BPA0.25, BPA2.5, BPA25 or BPA250) and sacrificed at date *k* (k=PND21, PND50, PND90, PND140 or PND200), *μ_j_* an overall constant, *α_kj_* the effect of the Time factor, *β_hj_* the effect of the Dose factor, *(αβ)_hkj_* the interaction of the Time and Dose factors, and *ε_hkij_* the residuals (variation not explained by the Time, Dose and Interaction factors).

Parameters of Equation 1 are estimated from means as follows:

*μ_j_ = Σ^5^_k=1_Σ^5^_h=1_Σ^nhk^_h=1_x_hkij,_ ∀ j = 1, …, J*

*α_kj_ = Σ^5^_h=1_Σ^nhk^_i=1_x_hkij,_ ∀ j = 1, … , J et ∀ h = 1, … , 5*

*β_hj_ = Σ^5^_k=1_Σ^nhk^_i=1_x_hkij,_ ∀ j = 1, … , J et ∀ k = 1, … , 5*

*(αβ)_hkj_ = Σ^nhk^_i=1_x_hkij,_ ∀ j = 1, … , J, ∀ h = 1, … , 5 et ∀ k = 1, … , 5.*

The *J* measures *x_hkij_* and the *J* parameter estimates can be collected in matrices. (Equation 1) which then becomes Equation 2:

*X = 1m’ + X_a_ + X_b_ + X_(ab)_.*

To deal with unbalanced data and ensure the independence of the estimation of the different effect (Time, Dose, Time*Dose), data were imputed for groups with n<10: missing data were randomly generated from normal distribution with mean and standard deviation calculated from observations within the corresponding groups. This independence of parameter estimates enables the subsequent individual analysis of each data block in Equation 2 by PCA. The Permutation test was used to test the significance of each experimental factor, and permits the validation of the model.

**PCA** is a projection method used to reduce data dimensionality. Linear combinations of NMR buckets are calculated by variation maximization. Weights of these linear combinations are included in the loading matrix P. Coordinates of individuals in the new variable space are in the scores matrix T. PCA decomposition is written as follows (Equation 3):

*X = TP’ + ε,*

where X is either X_a_, X_b_ or X_(ab)_.
